# Quantification of the Changes in Potent Wine Odorants as Induced by Bunch Rot (*Botrytis cinerea*) and Powdery Mildew (*Erysiphe necator*)

**DOI:** 10.3389/fchem.2017.00057

**Published:** 2017-08-03

**Authors:** Angela Lopez Pinar, Doris Rauhut, Ernst Ruehl, Andrea Buettner

**Affiliations:** ^1^Department of Chemistry and Pharmacy, Emil Fischer Center, Friedrich-Alexander-Universität Erlangen-Nürnberg Erlangen, Germany; ^2^Department of Microbiology and Biochemistry, Hochschule Geisenheim University Geisenheim, Germany; ^3^Department of Grapevine Breeding, Hochschule Geisenheim University Geisenheim, Germany; ^4^Department Sensory Analytics, Fraunhofer Institute for Process Engineering and Packaging IVV Freising, Germany

**Keywords:** Stable Isotope Dilution Analysis (SIDA), γ-decalactone, acetic acid, phenylacetic acid

## Abstract

Fungal infections are detrimental for viticulture since they may reduce harvest yield and wine quality. This study aimed to characterize the effects of bunch rot and powdery mildew on wine aroma by quantification of representative aroma compounds using Stable Isotope Dilution Analysis (SIDA). For this purpose, samples affected to a high degree by each fungus were compared with a healthy sample in each case; to this aim, the respective samples were collected and processed applying identical conditions. Thereby, the effects of bunch rot were studied in three different grape varieties: White Riesling, Red Riesling and Gewürztraminer whereas the influence of powdery mildew was studied on the hybrid Gm 8622-3. Analyses revealed that both fungal diseases caused significant changes in the concentration of most target compounds. Thereby, the greatest effects were increases in the concentration of phenylacetic acid, acetic acid and γ-decalactone for both fungi and all grape varieties. Regarding other compounds, however, inconsistent effects of bunch rot were observed for the three varieties studied.

## Introduction

*Botrytis cinerea* and *Erysiphe necator* are two fungi responsible for two vineyard diseases with high negative commercial impact: bunch rot (*B. cinerea*) and powdery mildew (*E. necator*). However, *B. cinerea* may also positively impact wine quality under special climatic conditions producing noble rot; in the wake of this process, highly valued botrytized wines are achieved.

Numerous studies previously addressed the impact of these fungi on wine quality in general, however, less work focused on wine aroma quality specifically. Overall, most literature addressed the aroma of the three most important examples of sweet botrytized wines: Sauternes (Bailly et al., 2006, 2009; Sarrazin et al., 2007; Campo et al., 2008), Amarone (Genovese et al., 2007; Fedrizzi et al., 2011; Tosi et al., 2012), and Tokaji Aszú (Schreier et al., 1976; Miklosy et al., 2000, 2004; Miklosy and Kerenyi, 2004). On the other hand, the effects of the negative form of *B. cinerea*, i.e., bunch rot and sour rot have been addressed in only a few investigations (Zoecklein et al., 2000; Meneguzzo et al., 2006; Barata et al., 2011; Ky et al., 2012). Thereby, generation of earthy off-odors has been described as consequence of bunch rot and has been attributed to the appearance of geosmin, 2-methylisoborneol and 1-octen-3-one, amongst others (La Guerche et al., 2005, 2006, 2007).

On the other hand, powdery mildew infection was studied in relation to wine quality by analyzing classic oenological parameters, namely harvest yield, total soluble solids, color and acidity, and/or performing sensory tests (Pool et al., 1984; Gadoury et al., 2001; Stummer et al., 2003, 2005; Calonnec et al., 2004). Thereby it was observed that this disease may cause a reduction in harvest yield, but also lower total soluble solids content, a less intense juice color and higher acidity have been reported as negative side-effects. Furthermore, in some studies, powdery mildew was related to the generation of off-notes that were described as earthy, dusty and reminiscent of mushroom (Darriet et al., 2002; Stummer et al., 2005). To the best of our knowledge, the effects of powdery mildew on wine aroma composition have been studied by Aroma Extract Dilution Analysis (AEDA) only once (Darriet et al., 2002). This study showed that two of the most important odorants in must, produced of diseased grapes, 1-octen-3-one and (*Z*)-1,5-octadien-3-one, were enzymatically reduced during (wine) fermentation to less odorous compounds, namely 3-octanone, and (*Z*)-5-octen-3-one. In addition, an unidentified fishy-mushroom-like smelling odorous zone was detected. However, a direct comparison between powdery mildew-affected samples and their respective healthy controls is, to the best of our knowledge, not yet reported in literature.

The aroma body constitutes the primary quality factor of wine. Consequently, it is important to understand the parameters that influence aroma composition of this highly valued product. Well-established analytical approaches are at hand in modern aroma analysis to relate the odorant composition of wine to its sensory profile: Gas Chromatography-Olfactometry (GC-O) techniques allow the screening for the aroma active constituents amongst a large number of volatile compounds and ranking according to their relative intensities as is done e.g., by AEDA (Grosch, 2001). This strategy is commonly complemented by sensory evaluations and quantification of the main odorous constituents, as well as reconstitution experiments, especially when the aim is the confirmation of the complete aroma spectrum.

This article is based on our previous study where effects of bunch rot and powdery mildew on wine aroma have been evaluated by comparative AEDA and sensory tests (Lopez Pinar et al., 2017). Thereby, the aim of the present study is to validate the olfactometric observations by means of quantitative determinations.

## Materials and methods

### Chemicals

Dichloromethane and anhydrous sodium sulfate were purchased from VWR (Darmstadt, Germany) whereas sulfuric acid and sodium carbonate were from Roth (Karlsruhe, Germany). The reference substances were obtained from the following suppliers: acetic acid, 2-phenylethanol, 3-methyl-1-butanol (isoamyl alcohol), hexanoic acid, phenylacetic acid, ethyl 2-methylbutanoate, γ-decalactone, γ-undecalactone, 2-methylbutanoic acid and 3-(methylthio)-propanal (methional) were from Sigma-Aldrich (Steinheim, Germany), butanoic acid and ethyl butanoate from Fluka (Steinheim, Germany), 2-methylpropanoic acid (isobutanoic acid) from SAFC (Steinheim, Germany), and 4-hydroxy-3-methoxybenzaldehyde (vanillin) from ABCR (Karlsruhe, Germany). The isotopically labeled standards were purchased from the following suppliers given in parentheses: 3,4-^2^H_2_-3-methyl-1-butanol (^2^H_2−_isoamyl alcohol), 1,3,4,5,6-^2^H_5_-2-phenylethanol, 3-(methyl-^2^H_3_-thio)-propanal (^2^H_3_-methional), 5,5,6,6-^2^H_4_-y-decalactone, 6,7-^2^H_2_-y-undecalactone (Aromalab, Freising, Germany), 6,6,6-^2^H_3_-hexanoic acid, 3,3,3-^2^H_3_-2-methylpropanoic acid (^2^H_3_-isobutanoic acid), and ^2^H_6_-ethanol (CDN Isotopes, Pointe-Claire, Quebec, Canada), 1,2-^13^C_2_-butanoic acid, α,β-^13^C_2_–phenylacetic acid, ^13^C_1_-acetic acid, and 4-hydroxy-3-methoxy-^13^C_6−_benzaldehyde (^13^C_6_-vanillin) (Sigma-Aldrich, Steinheim, Germany).

### Synthesis of ^2^H_5_-ethyl butanoate and ^2^H_5_-ethyl 2-methylbutanoate

^2^H_5_-Ethyl butanoate and ^2^H_5_-ethyl 2-methylbutanoate were synthesized in the following way: [^2^H_6_]-ethanol (25 mmol, 1.30 g) and concentrated sulfuric acid (1 mmol, 0.098 g) were mixed with either butanoic acid (5 mmol, 0.44 g) to give ^2^H_5_-ethyl butanoate or 2-methylbutanoic acid (5 mmol, 0.51 g) to give ^2^H_5_-ethyl 2-methylbutanoate; the mixtures were stirred at 90°C for 2 h. To obtain the final products, 50 mL of sodium carbonate water solution (0.5 mol/L) were added, and the resulting mixtures were extracted thrice with 50 mL of dichloromethane. After vigorous shaking, the organic phase was separated from the aqueous phase using a separatory funnel. The combined organic phases were dried over anhydrous sodium sulfate. The products were concentrated using a R-114 rotary evaporator (Buechi, Essen, Germany) without application of vacuum at 50°C to a final volume of approximately 50 mL. The identity of the synthesized compounds was confirmed by mass spectrometry. For ^2^H_5_-ethyl butanoate the following data was obtained: MS-EI: 43 (100), 71 (91), 93 (68), 41 (49), 39 (48), 42 (41), 74 (31), 40 (27), 91 (23), 106 (15), 92 (14), 67 (12), 56 (11), 69 (11); MS-CI: 122 (100, M^++1^), 121 (18), 71 (10). For ^2^H_5_-ethyl 2-methylbutanoate the following data was obtained: MS-EI: 107 (100), 57 (87), 41 (48), 75 (37), 39 (26), 88 (19), 56 (18), 120 (17), 76 (12); MS-CI: 136 (100, M^++1^), 135 (21).

### Wine samples

The effects of bunch rot were studied in three grape varieties: White Riesling, Red Riesling and Gewürztraminer. In the case of powdery mildew, the hybrid Gm 8622-3 was analyzed. Sample selection and grape harvest procedures have already been described in our previous study (Lopez Pinar et al., 2016). In brief, samples affected by the fungus to a very high degree were compared with their respective healthy control. In each case, the pairs of samples were collected separately, but both were harvested and processed identically. The degree of fungal infection of the grape berries was visually assessed as described in our previous study (Lopez Pinar et al. (2016), providing photographs with representative examples of grapes affected with bunch rot and powdery mildew). Grapes were crushed, pressed in a pneumatic press and left to settle with 50 mg/L SO_2_ for approximately 24 h at 10°C, followed by filtering through filter paper. The grape musts were subsequently fermented at 17°C for 2–3 weeks in 20 L glass balloons by inoculation of the reactivated pure yeast culture (20 g/hL) Lalvin EC 1118 (Lallemand, Vienna, Austria). After fermentation, wines were racked and the content of SO_2_ was adjusted to achieve a final concentration of approximately 50 mg/L free SO_2_. Finally, samples were filled in brown glass bottles which were closed with screw caps. The wines were stored for approximately 1 year at room temperature. Residual sample material from opened bottles was kept at −80°C if required.

### Determination of basic oenological parameters

Residual sugar, titratable acidity and alcoholic content (v/v) were determined using a Winescan FT-2 Fourier Transform Infrared Spectrometer (FTIR) (Foss, Hillerød, Denmark) as described previously (Patz et al., 1999, 2004).

### High resolution gas chromatography–mass spectrometry (GC–MS)

Mass spectra were recorded using a 5975C MSD quadrupole system combined with a 7890A GC system (Agilent Technologies, Waldbronn, Germany). The instrument was equipped with a Gerstel CIS 4 injection system and a Gerstel MPS 2 auto sampler (Gerstel, Duisburg, Germany), and a DB-FFAP (30 m, 0.25 mm, film thickness 0.25 μm; J&W Scientific, Fisons Instruments, Mainz-Kastel, Germany) as the analytical capillary column. An uncoated, deactivated fused silica capillary column was used as precolumn (2–3 m, 0.53 mm). The carrier gas was helium and the flow rate was 1 mL/min. The temperature program used was as follows: the initial 40°C were held for 2 min, then the temperature was raised at 8°C/min until 240°C, and held for 5 min. Injection volume was 1 μL. EI-mass spectra were generated in full scan mode (m/z range 40–400) at 70 eV ionization energy. The scan rate was 3.94 scan/s.

### Two-dimensional high resolution gas chromatography–mass spectrometry/olfactometry (HRGC–GC–MS/O)

Two-dimensional gas chromatographic analyses were carried out for quantification of trace constituents and specifically for those compounds that could not be fully resolved due to interferences from coeluting substances during one dimensional GC-MS analysis (Table 1). The system consisted of two Varian 450-GCs (Varian, Darmstadt, Germany) combined with a Saturn 2200 MS (Varian). The first GC was equipped with a Gerstel multi-column switching system MCS 2 and connected to the second GC by a Gerstel cryo-trap system CTS 1 (Gerstel). The system was further equipped with a Gerstel CIS 3 injection system and Gerstel MPS 2 auto sampler (Gerstel). The analytical capillaries used were a DB-FFAP capillary (30 m, 0.32 mm, film thickness 0.25 μm; J&W Scientific) in the first oven and a Rxi®-5HT (30 m, 0.25 mm, film thickness 0.25 μm; Restek, Homburg, Germany) in the second oven. At the end of the capillary, the effluent was split between a sniffing port and a FID, or a MS, in the first and second oven, respectively, using two deactivated but uncoated fused silica capillaries (100 cm, 0.20 mm). Application of the samples to the GC system was performed at 40°C using the cold-on-column technique. The GC temperature programs were as follows: the initial 40°C were held for 2 min, then the temperature was raised at 8°C/min until 240°C (for DB-FFAP measurements) or 250°C (for Rxi®-5HT measurements) were reached; the final temperature was then held for 5 min. The flow rate of the helium carrier gas was 2.0 mL/min. The FID and the sniffing port were held at 240 and 260°C, respectively. Mass spectra were generated at 70 eV ionization energy in electron ionization (EI) mode (m/z range 30–300) or in the positive chemical ionization (CI) mode with methanol as the reactant gas (m/z range 35–300).

**Table 1:** Parameters of the quantification method including isotopically labeled standards, selected ions, calibration factors, ionization mode, instruments on which the measurements were performed and volume (ml) of wine extracted to quantify the respective aroma compounds.

**Measurements^a^**	**Ionization mode**	**Volume wine extracted (ml)^b^**	**Odorant**	**Ion m/z**	**Internal standard**	**Ion m/z**	**Calibration line equation**	**R^2^**
GC-MS	EI	1	Acetic acid	60	^13^C_1_-Acetic acid	61	*y* = 0.9354x + 0.0687	0.999
			2-Phenylethanol	91	1,3,4,5,6-^2^H_5_-2-Phenylethanol	96	*y* = 2.1240x + 0.0371	0.996
			3-Methyl-1-butanol (Isoamyl alcohol)	70	3,4-^2^H_2_-3-Methyl-1-butanol	72	*y* = 2.0352x + 0.4604	0.995
		5	Butanoic acid	73	1,2-^13^C_2_-Butanoic acid	75	*y* = 7.3461x + 0.8346	0.999
			Hexanoic acid	99	6,6,6-^2^H_3_-Hexanoic acid	102	*y* = 0.9377x + 0.1185	0.995
			2-Methylpropanoic acid (Isobutanoic acid)	88	3,3,3-^2^H_3_-2-Methylpropanoic acid	91	*y* = 1.1928x − 0.0988	0.999
		60	Ethyl butanoate	88	1,1,2,2,2-^2^H_5_-Ethyl butanoate	93	*y* = 0.83x + 0.067	0.996
			Phenylacetic acid	136	α,β-^13^C_2_–Phenylacetic acid	138	*y* = 0.9327x − 0.0176	0.999
2 DIM GC-MS	EI		4-Hydroxy-3-methoxybenzaldehyde (Vanillin)	151	4-Hydroxy-3-methoxy-^13^C_6_-benzaldehyde	157	*y* = 0.964x − 0.005	0.998
			Ethyl 2-methylbutanoate	102	1,1,2,2,2-^2^H_5_-Ethyl 2-methylbutanoate	107	*y* = 1.081x + 0.1283	0.994
	CI		3-(Methylthio) propanal (Methional)	105	3-(Methyl-^2^H_3_-thio) propanal	108	*y* = 0.8634x + 0.0659	0.998
			γ-Decalactone	171	5,5,6,6-^2^H_4_-γ-Decalactone	175	*y* = 1.5782x − 0.4605	0.996
			γ-Undecalactone	167	6,7-^2^H_2_-γ-Undecalactone	169	*y* = 1.1267x + 0.0478	0.991

a *Instruments on which the measurements for the quantifications were performed*.

b *Volume (ml) of wine extracted to quantify the respective aroma compounds*.

### Quantification by means of stable isotope dilution analysis (SIDA)

In the first step, the isotopically labeled compounds dissolved in dichloromethane were mixed to the wine samples containing the odorant at comparable concentrations, as determined in preliminary experiments. Volumes of wines employed in the analyses were 1, 5, and 60 mL, respectively, depending on the concentrations of the compounds in the wine samples (cf. Table 1). For subsequent extraction of the volatile compounds, wine samples were mixed with dichloromethane in the ratio 1:1 (v/v), or 1:5 (v/v) in case that the volume of extracted wine was only 1 mL. These mixtures were stirred for 1 h at room temperature, and then submitted to solvent assisted flavor evaporation (SAFE) (Engel et al., 1999). The obtained dichloromethane phases were separated in each case, and dried over anhydrous sodium sulfate. Finally, each extract was concentrated to a final volume of 100 μL at 51°C by means of Vigreux distillation followed by micro-distillation.

The selected ions of the labeled and unlabeled compounds were monitored by one- or two-dimensional GC-MS (cf. Table 1), and their intensities recorded in relation to each other. The absolute concentrations were then calculated based on these relative intensities and the amount of labeled standard that had been added in each case, and were corrected by using calibration factors obtained from the mixtures of labeled and unlabeled compounds (cf. Table 1) as described in Spitzer et al. (2013). Analyses were performed per triplicate in each case.

### Statistical analyses

The statistical significance of the effects of the two fungal diseases on the concentrations of the target aroma compounds was analyzed based on a comparison of the mean quantitative values from affected and unaffected samples using Student's *t*-test. Data were evaluated with regard to a significance level of *p* < 0.05 and *p* < 0.01.

## Results

### Basic oenological parameters

Three basic oenological parameters were analyzed: residual sugar, titratable acidity and alcoholic percentage (Table 2).

**Table 2:** Some basic oenological parameters of the wine samples analyzed in the current study: grape varieties were White Riesling, Red Riesling, Gewürztraminer and the hybrid Gm 8622-3; grapes were grouped according to their health status in three groups: completely healthy, minor fungus infection and infected by Botrytis bunch rot or Powdery mildew, respectively.

		**Sample**		**Residual sugar (g/L)**	**Titratable acidity (g/L)**	**Alcohol (% v/v)**
Bunch rot	White Riesling	Healthy	HW	1.0	10.2	11.6
		Intermediate state	IW	5.9	9.3	13.9
		Bunch rot affected	BW	6.4	9.1	13.6
	Red Riesling	Healthy	HW	1.8	10.4	11.3
		Bunch rot affected	BW	3.8	9.4	13.6
	Gewürztraminer	Healthy	HW	1.8	7.5	11.8
		Bunch rot affected	BW	1.3	8.3	12.4
Powdery mildew	Gm 8622-3	Healthy	HW	10.4	9.2	12.9
		Powdery mildew affected	PW	5.0	8.1	13.5

*The presented data are the result of one single measurement*.

Residual sugar was higher in the sample infected by bunch rot than in the corresponding healthy sample of the White Riesling and Red Riesling varieties, but in Gewürztraminer, residual sugar was moderately lower in the affected sample. In contrast to this, a much lower level of residual sugar was observed in the powdery mildew-affected sample.

Titratable acidity decreased moderately in the samples affected by bunch rot for White Riesling and Red Riesling. However, the Gewürztraminer sample again showed a different tendency with titratable acidity being higher in the affected sample. On the other hand, acidity in the powdery mildew-affected sample was lower than in the healthy sample.

Finally, the alcohol level was higher in all four fungi-affected samples in comparison to their respective healthy samples.

### Quantification

In order to characterize the effects of both fungi on the concentration of the selected aroma compounds, wine samples affected to a high degree by bunch rot (BW) or powdery mildew (PW) were compared with healthy ones (HW) of the same variety. Each pair of samples was collected and processed under identical conditions. In case of White Riesling, an additional intermediate state of bunch rot-infection (IW) was included (Table 3). Target odorants were selected based on our previous study where the main changes in odorant profile with fungi infection were elaborated by means of aroma extract dilution analyses and odorant identification studies (Lopez Pinar et al., 2017). Thereby, marker substances representative for acids, esters, alcohols, lactones and aldehydes were selected.

**Table 3:** Results of the quantification experiments.

	**Units of concentration**	**Bunch rot effects**							**Powdery mildew effects**	
		**White Riesling**			**Red Riesling**		**Gewürztraminer**		**Gm 8622-3**	
		**HW**	**IW**	**BW**	**HW**	**BW**	**HW**	**BW**	**HW**	**PW**
3-Methyl-1-butanol (Isoamyl alcohol)	μg/g	203.0 ± 8.7	195.7 ± 18.1	202.9 ± 13.8	213.1 ± 20.0	142.1 ± 11.1^**^	195.1 ± 20.4	130.8 ± 8.6^*^	205.6 ± 16.4	201.7 ± 20.4
Acetic acid		404.0 ± 7.4	474.1 ± 20.6^*^	666.7 ± 9.0^**^	443.8 ± 7.8	861.1 ± 21.1^**^	346.9 ± 3.4	726.3 ± 66.8^*^	285.0 ± 5.4	314.9 ± 8.4^*^
2-Phenylethanol		32.8 ± 0.8	29.3 ± 4.4	41.4 ± 2.1^*^	35.8 ± 2.6	28.7 ± 3.7	14.2 ± 0.3	12.0 ± 0.3^**^	19.3 ± 0.2	23.6 ± 0.3^**^
Hexanoic acid		0.9 ± 0.1	0.8 ± 0.1	1.1 ± 0.2	1.7 ± 0.2	1.2 ± 0.3^*^	2.1 ± 0.3	1.3 ± 0.5	1.8 ± 0.4	1.9 ± 0.2
Butanoic acid		0.2 ± 0.0	0.2 ± 0.1	0.2 ± 0.0	0.2 ± 0.0	0.2 ± 0.0	0.2 ± 0.0	0.1 ± 0.0^**^	0.2 ± 0.0	0.2 ± 0.0
2-Methylpropanoic acid (Isobutanoic acid)		1.3 ± 0.1	2.2 ± 0.2^*^	2.3 ± 0.3^*^	1.3 ± 0.0	2.1 ± 0.2^*^	1.7 ± 0.1	2.7 ± 0.1^**^	1.9 ± 0.1	2.6 ± 0.0^**^
Ethyl butanoate		0.2 ± 0.0	0.2 ± 0.1	0.2 ± 0.1	0.2 ± 0.0	0.3 ± 0.0^**^	0.3 ± 0.0	0.2 ± 0.0^**^	0.3 ± 0.0	0.3 ± 0.0
Phenylacetic acid	μg/kg	8.3 ± 0.3	9.9 ± 1.1	21.6 ± 0.6^**^	9.9 ± 1.6	21.5 ± 1.9^**^	11.9 ± 0.8	32.5 ± 0.3^**^	1.6 ± 0.0	8.6 ± 0.3^**^
γ-Decalactone		1.3 ± 0.3	0.6 ± 0.0	1.0 ± 0.0	0.8 ± 0.1	3.9 ± 0.1^**^	0.9 ± 0.0	2.5 ± 0.2^**^	0.3 ± 0.0	0.8 ± 0.1^**^
γ-Undecalactone		0.2 ± 0.0	0.2 ± 0.0	0.2 ± 0.1	0.1 ± 0.0	0.2 ± 0.0	0.1 ± 0.0	0.1 ± 0.0	0.1 ± 0.0	0.1 ± 0.0
Ethyl 2-methylbutanoate		17.8 ± 2.7	18.6 ± 1.2	11.6 ± 1.9^*^	11.9 ± 3.6	4.8 ± 0.1	3.8 ± 0.2	3.8 ± 0.3	7.3 ± 0.6	7.5 ± 0.2
4-Hydroxy-3-methoxy benzaldehyde (Vanillin)		3.0 ± 0.8	2.4 ± 0.3	2.7 ± 0.7	3.4 ± 0.9	4.0 ± 0.4	2.3 ± 0.1	5.6 ± 0.6^**^	2.2 ± 0.3	3.3 ± 0.4
3-(Methylthio)-propanal (Methional)		1.7 ± 0.3	1.2 ± 0.3^*^	1.7 ± 0.4	2.2 ± 0.4	0.8 ± 0.1^*^	1.3 ± 0.0	0.9 ± 0.1^*^	0.4 ± 0.0	1.0 ± 0.1^**^

*Comparison of healthy samples with samples affected by bunch rot or powdery mildew, respectively. Bunch rot effects were investigated in White Riesling, Red Riesling and Gewürztraminer, whereas powdery mildew effects were studied in the hybrid Gm 8622-3*.

*The results were obtained from independent triplicate treatments*.

* *Concentration of the substance in the respective bunch rot or powdery mildew-affected sample was significantly different (p < 0.05) in comparison to the corresponding healthy sample*.

** *Differences between bunch rot or powdery mildew-affected sample and the corresponding healthy samples were highly significant (p < 0.01)*.

First, quantification experiments revealed that the most abundant odorants in the wine samples were isoamyl alcohol and acetic acid: both compounds were found in all samples at concentrations between 100 and 900 μg/g.

In the following, fungi effects on the respective odorant concentrations are presented. First and foremost, concentrations of phenylacetic acid and γ-decalactone were significantly increased (*p* < 0.01) by both fungi. Thereby, their concentrations in the fungi-infected samples increased by a factor of ~2–5 in comparison to the corresponding healthy samples. White Riesling was the sole exception where no significant change in γ-decalactone concentration was observed. In addition, acetic acid roughly doubled in concentration, in case of bunch rot infection (*p* < 0.05) whereas powdery mildew effects were only moderate (< factor 1.5).

Apart from that, vanillin concentration doubled in the Gewürztraminer bunch rot affected sample (*p* < 0.01). In contrast, its concentration remained unaffected in White Riesling and Red Riesling varieties and in the sample affected by powdery mildew.

In addition, the concentrations of methional were differently affected by both fungal infections. It was decreased (*p* < 0.05) by bunch rot in Red Riesling and Gewürztraminer, being lower by a factor of about 1.5–3. In contrast to this, its concentration was not affected in White Riesling variety. On the other hand, the concentration of methional doubled due to powdery mildew infection (*p* < 0.01).

The concentrations of several other compounds were also affected by both fungal diseases, but only moderately. Thereby, the changes described below were smaller than a factor of 2.

In the group of the cheesy smelling acids, inconsistent effects were recorded. Isobutanoic acid concentrations were higher (*p* < 0.05) in all four fungi-affected samples than in their corresponding healthy samples. In contrast to this, hexanoic acid concentrations decreased (*p* < 0.05) due to bunch rot infection, however, only in Red Riesling variety. Powdery mildew did not cause any significant effects on the concentration of this acid. Likewise, bunch rot induced a decline (*p* < 0.01) in butanoic acid in Gewürztraminer. Meanwhile, butanoic acid concentration was neither affected in the remaining bunch rot-affected samples nor in the sample affected by powdery mildew.

In the group of alcohols, isoamyl alcohol concentrations were lower (*p* < 0.05) in the Red Riesling and Gewürztraminer samples affected by bunch rot than in the corresponding healthy samples. In the White Riesling bunch rot-affected sample, however, the concentration did not vary from that in the healthy sample or in the sample infected to an intermediate state. Lastly, powdery mildew infection did not lead to any relevant differences in the concentration of this compound. Bunch rot effects on the concentration of 2-phenylethanol differed among the three varieties studied: it was increased (*p* < 0.05) in the White Riesling bunch rot-affected sample, unchanged in Red Riesling, and decreased (*p* < 0.01) in Gewürztraminer. In addition, a significant rise (*p* < 0.01) in concentration of this substance occurred in case of powdery mildew infection.

With regard to the ester substance group, variable results were obtained. Bunch rot led to an increase (*p* < 0.01) of ethyl butanoate in Red Riesling, a decrease (*p* < 0.01) in Gewürztraminer, whereas its concentration in White Riesling was not affected. Powdery mildew, on the other hand, caused no significant changes in the concentration of this compound. In addition, bunch rot caused a significant decrease (*p* < 0.05) of ethyl 2-methylbutanoate in White Riesling. However, significant effects on the concentration level of this substance were neither observed in the remaining varieties nor in the sample affected by powdery mildew.

Finally, γ-undecalactone was neither affected by bunch rot nor by powdery mildew.

## Discussion

The influence of bunch rot on wine oenological parameters differed among the grape varieties studied: in Gewürztraminer bunch rot-affected sample, residual sugar was lower and titratable acidity higher than in the corresponding healthy sample. White and Red Riesling, however, showed the opposite trend. Moreover, alcoholic contents were higher in the samples affected by bunch rot in all three varieties, but the increase was less pronounced in Gewürztraminer. These variations may be directly linked to the different levels of total soluble solids (°Brix) in the corresponding grape musts (Lopez Pinar et al., 2016): in White and Red Riesling, °Brix were increased due to bunch rot infection by 25.6 and 26.7%, respectively. In Gewürztraminer °Brix only increased by 7.7%.

On the other hand, bunch rot and powdery mildew infection induced significant changes in the concentrations of most studied aroma compounds; nevertheless the majority of these changes was only in a range of about a factor 1.5–2.

Both fungal diseases caused a significant increase in acetic acid concentrations; thereby, bunch rot effects were more pronounced than the changes induced by powdery mildew. Generally, the content of this compound increases with the degree of oxidation and is also related to a higher production of the oxidative enzyme laccase induced by *B. cinerea* (Slomczynski et al., 1995; Pezet, 1998). Additionally, bunch rot and powdery mildew infected grapes are susceptible to secondary infections (Steel et al., 2013), such as acetic acid bacteria, which convert ethanol into acetic acid (Cleenwerck and De Vos, 2008; Raspor and Goranovič, 2008).

Moreover, isobutanoic acid concentration was increased in all fungi-affected samples. This compound is formed by deamination of branched amino acids (Lilly et al., 2006), being a metabolic strategy of the fungi to utilize nitrogen for their metabolism (Mencarelli and Tonutti, 2013). The same mechanism might be related to the remarkable rise in phenylacetic acid levels, which is likely to be related to the deamination of phenylalanine (Somers et al., 2005). Moreover, the higher content of phenylacetic acid may be part of a defense mechanism of the plant against fungal infections since this compound is known to be an auxin with a reported antifungal activity (Wightman and Lighty, 1982; Hwang et al., 2001). Indeed, it has been reported that fungal infections may cause a rise in auxin levels (Hare, 1966).

In addition, concentration of γ-decalactone increased significantly due to bunch rot and powdery mildew infection, being in agreement with previous reports of an increase of lactones caused by noble rot, thereby playing an important role in the aroma of the so called botrytized wines (Schreier et al., 1976; Miklosy and Kerenyi, 2004; Miklosy et al., 2004). However, an increase of lactones due to powdery mildew infection has, to the best of our knowledge, not been previously reported. On the other hand, the level of γ-undecalactone was not affected. The reasons for this differential effect are not clear as such pattern has not been reported before, but might be related to a differential metabolism of lactones with even carbon atom numbers.

Bunch rot effects on several aroma compounds from the esters and alcohols group varied among the varieties studied. These divergences cannot be directly related to simple relationships. Indeed, the formation of alcohols and esters in the course of fermentation is influenced by a complex combination of grape composition, yeast metabolism and winemaking conditions (Carrau et al., 2010; Antalick et al., 2015). Yeast strain and fermentation conditions were the same for all varieties in the present study; accordingly, the differences can only be due to different metabolic processes or differences in general infection susceptibility of the investigated varieties. Furthermore, bunch rot is mainly related to *B. cinerea*, but there might be other secondary infections involved (Steel et al., 2013). In this way, possible differences in the microbial flora may have caused these variations. Surely, this issue should be investigated in more detail in future studies.

To summarize our findings, this study aimed to complement the results obtained by olfactometric evaluations as reported in our previous publication (Lopez Pinar et al., 2017). Accordingly, the most relevant outcomes are briefly summarized in the following. The greatest effects were an increase of the concentrations of acetic acid, phenylacetic acid and γ-decalactone in the bunch rot-affected samples, which confirms the corresponding increases in their FD (flavor dilution) factors as observed via AEDA in our previous study. The concentration of phenylacetic acid was also increased by powdery mildew to a relevant extent which is in line with a higher FD factor in the affected sample. In comparison to that, y-decalactone concentration only roughly doubled due to powdery mildew infection, and this change was not mirrored in a relevant change in FD factor as it was not sufficiently pronounced. Commonly it needs to be stated that AEDA is an olfactory screening approach that may be prone to some variation, commonly in the range of about one or sometimes even two FD factors between individual sample runs. Accordingly, this example clearly demonstrates that AEDA can never fully replace absolute quantification experiments. Apart from that, aroma compounds may result in rather unpredictable sensory effects when being combined in mixtures and different ratios. Additive, synergistic and suppressive effects together with complex peripheral and perceptual modulation and processing steps may lead to sensory perceptions that are, at times, hard to directly correlate with the respective underlying odorants (Buettner and Beauchamp, 2010). Accordingly, future studies need to expand on these aspects if aiming at gaining a fundamental understanding of aroma changes induced by microbial processes as described in the current study.

## Conclusion

Quantification of representative odor compounds confirmed the influence of bunch rot and powdery mildew on wine aroma. The main differences in the concentration of individual marker substances corresponded with differences as observed in the previous AEDA experiments (Lopez Pinar et al., 2017).

The most important changes caused by both types of infections were increases in the content of acetic acid, phenylacetic acid and γ-decalactone. Moreover, the quantification experiments confirmed the divergent effects of bunch rot on several other aroma compounds from the substance groups of alcohols and esters in the three varieties studied.

Future studies should address the potential influence of secondary infections and the impact of varying climatic conditions. In addition, it should be addressed how the specific susceptibility of a specific variety influences the degree of infection in relation to the associated changes in aroma and flavor profile.

## Author contributions

Each author has participated sufficiently in the work, intellectually or practically, to take public responsibility for the content of this article, including the conception, design, and conduct of the experiment and data analysis and interpretation. AL, DR, and ER were responsible for sampling. AL, DR, and ER carried out the practical work. AL, DR, and ER were responsible for data analysis. AB, AL, DR, and ER conceived the study, and AB, AL, DR, and ER participated in the design of the study. All authors contributed to the manuscript and approved the final version.

### Conflict of interest statement

The authors declare that the research was conducted in the absence of any commercial or financial relationships that could be construed as a potential conflict of interest. The reviewer YL and handling Editor declared their shared affiliation, and the handling Editor states that the process nevertheless met the standards of a fair and objective review.
